# The Impregnation of the Graafian Vesicle

**Published:** 1857-08

**Authors:** G. S. Dewey

**Affiliations:** Ill.


### ARTICLE V.—*The Impregnation of the Graafian Vesicle.* By G. S. Dewey, M. D., Ill.

Electricity, or a fluid bearing a close resemblance to it in all its manifestations and regulations, is admitted to be the immediate efficient cause of vital phenomena.

It has been shown that this fluid, sent along the nerves of a dead body, excites muscular action. "The brain of a newly-killed animal being taken out, and replaced by a substance which produced electric action, the operation of digestion, which had been interrupted by the death of the animal, was resumed, showing the absolute identity of the brain with a galvanic battery."

The brain is composed of two physically different substances —the cineritious and medullary—which may bear to each other a similar relation to the two metallic plates, by which relation the nervous fluid is eliminated.

Thus similar are many of the operations of nature, insomuch that whenever we gain the hand of analogy to lead us through her dimly-lighted labyrinths, we feel a confidence and satisfaction which we derive from no other guide.

We may presume the male affords the positive and the female the ovum containing (with the pabulum to sustain it until it is provided for by the uterus) the opposite material. By copulation, these plates or opposite electrics are attached, and a galvanic current established, first at the fimbriated extremity of the fallopian tube, and carried on to the building up of the fœtus; with all the phenomena of life in the uterus, when it has melted away the ovisac, and by the peristaltic action of the tube being brought to that organ.

It is not necessary, in support of this hypothesis, to show a resemblance in masculine serum, or in the germinal vesicle of the female ovum, and their typical portions of the brain, for we know that very different substances may act upon each other to produce an electric current; but it is a singular coincidence that such a resemblance does really exist. Dunglison's Human Physiology, page 331, says: "The spine is regarded by some physiologists as formed of the most animalized material, or of those that constitute the most elevated part of the new being—the nervous system."

It is wisely diluted with albuminous fluid, to facilitate its primary wandering mission; yet the words in Carpenter's Human Physiology used to describe it would equally apply to the cineritious portion of the brain—"thick, tenacious, and of a *grayish*, yellow color." Their chemical composition is strikingly similar. The vittellus of the female ovum "is composed of albumen and oil particles, with traces of cells," and also bears a physical resemblance to its prototype.

These materials furnished by the opposite sex commence to perform the functions of the brain—assimilation and nutrition—the moment they are placed in juxtaposition, and we cannot well avoid the inference that it is by the same electric agent. They seem, therefore, to form a miniature brain, which is probably formed of *medullary* and *cineritious* matter.

No theory of fecundation is at all satisfactory which will not account for the resemblance of the offspring with the parents, by actual contribution of the peculiar organization of each—analagous is the contribution and stigma in amalgamating contiguous varieties of plants; and no supposition seems so reasonable as the mutual contribution to the embryo of the nervous matter which presides over its development.

I have not attempted to bring up the facts and analogies which favor this hypothesis; this would occupy too much space; but merely throw it out as a suggestion to be improved or rejected by those who rule the province of physiology.

